# Successful Transmammary Treatment of *Babesia gibsoni* Infection in Newborn Puppies after the Administration of Malarone^®^, Azithromycin, and Artesunate to a Lactating Dam

**DOI:** 10.3390/pathogens13070562

**Published:** 2024-07-03

**Authors:** Martina Karasová, Lucia Blaňarová, Csilla Tóthová, Gabriela Hrčková, Terézia Kisková, Zuzana Ševčíková, Viera Revajová, Oskar Nagy, Bronislava Víchová

**Affiliations:** 1 Small Animal Clinic, University of Veterinary Medicine and Pharmacy, 04001 Košice, Slovakia; 2 Department of Clinical Microbiology, St. Jakub’s Hospital with Polyclinic, 08501 Bardejov, Slovakia; luciablanarova@nsp-bardejov.sk; 3 Clinic of Ruminants, University of Veterinary Medicine and Pharmacy, 04001 Košice, Slovakia; csilla.tothova@uvlf.sk (C.T.); oskar.nagy@uvlf.sk (O.N.); 4 Laboratory of Experimental Pharmacology, Institute of Parasitology, Slovak Academy of Sciences, 04001 Košice, Slovakia; hrcka@saske.sk; 5 Department of Animal Physiology, Faculty of Science, University of Pavol Jozef Šafárik, 04180 Košice, Slovakia; terezia.kiskova@upjs.sk; 6 Department of Morphological Disciplines, University of Veterinary Medicine and Pharmacy, 04001 Košice, Slovakia; zuzana.sevcikova@uvlf.sk (Z.Š.); viera.revajova@uvlf.sk (V.R.); 7 Laboratory of Molecular Ecology of Vectors, Institute of Parasitology, Slovak Academy of Sciences, 04001 Košice, Slovakia; vichova@saske.sk

**Keywords:** artesunate, azithromycin, *Babesia gibsoni*, lactation, Malarone^®^, puppies

## Abstract

*Babesia gibsoni* is a parasitic protozoan transmitted through tick bites and can cause severe disease in dogs. It can also be transmitted through direct contact with infected blood during dog fights, blood transfusions, and from dam to offspring during the perinatal period, resulting in stillborn or dead newborn puppies. This study aimed to determine the incidence of infection, the viability of newborn puppies, and the degree of *B. gibsoni* transmission from infected dam to offspring during pregnancy and lactation. Using PCR-based molecular methods, *B. gibsoni* infection in a pregnant American Pit Bull Terrier and her newborn puppies was confirmed. The incidence of *B. gibsoni* infection in the litter reached 75%. Out of eight puppies, six were infected with *B. gibsoni*, and one died. A therapeutic protocol comprising Malarone^®^, azithromycin, and artesunate was administered to a lactating *B. gibsoni*-positive bitch. By day 77 after birth, three out of five positive puppies showed negative PCR tests for *B. gibsoni*, indicating successful treatment through breast milk during nursing. In the two remaining positive puppies, therapy was started and parasitemia was successfully eliminated.

## 1. Introduction

*Babesia gibsoni* is an emerging blood parasite responsible for canine babesiosis, a severe disease affecting domestic and wild canines worldwide [1,2,3]. Despite its relatively small size, this parasite has posed significant challenges to veterinary medicine and animal welfare, causing severe health issues in dogs, including anemia, fever, lethargy, and even death if left untreated [4]. The disease may follow various courses depending on the parasite strain and the animal’s age, immunological status, breed, origin, and co-morbidities [5].

Originating from Southeast Asia, *B. gibsoni* has spread globally due to the movement of infected animals [6]. The history of *B. gibsoni* dates to the early 20th century when it was first identified in the blood of infected dogs. Since then, extensive research has been conducted to understand its biology, transmission, and control measures [1,3,4].

In endemic areas, the transmission of *B. gibsoni* typically occurs through the bites of infected ticks of the genera *Rhipicephalus*, *Haemaphysalis*, and *Dermacentor* [7,8,9]. Moreover, the risk of transmission in dogs through direct contact with infected blood, often from dog fight bites or blood transfusions, is relatively high [10]. These transmission routes are particularly significant in predisposed breeds, such as American Pit Bull Terriers, Tosa Inu, or American Staffordshire Terriers, which are more susceptible to *B. gibsoni* infection [11,12].

Additionally, transplacental transmission occurs from infected bitches to puppies resulting in neonatal babesiosis, which can be severe and even life-threatening if not treated promptly [13]. The transplacental transmission of *B. gibsoni* was confirmed for the first time in an experimentally infected beagle female 650 days before mating with an intact male. The female showed a chronic course of the disease with mild clinical manifestations and intraerythrocytic parasitemia in the peripheral blood. There was one stillborn puppy in the litter and four puppies that died on days 14–19 after birth. All puppies suffered from hypothermia and anemia, and fell into a comatose state before their death. An autopsy confirmed splenomegaly and hepatomegaly. In all puppies, DNA analysis revealed the presence of *B. gibsoni*, which confirms the transplacental transmission of the blood parasites. The presence of *B. gibsoni* was confirmed in samples taken from the stillborn puppy’s brain, heart, lungs, and liver. Immediately after birth, the infected puppies were exchanged for puppies of another intact dam. In these puppies, the presence of *B. gibsoni* was not detected after the examination, which confirms that *B. gibsoni* is not transmitted via milk from a positive dam to puppies [13]. Several other studies also describe clinical cases of vertical transmission of *B. gibsoni* in dogs [14,15,16].

To determine the incidence of infection, the viability of the newborn pups, and the degree of vertical (transplacental) transmission of *B. gibsoni* from dam to offspring, the current study set out to describe a clinical case of a pregnant American Pit Bull Terrier female affected by the parasite during pregnancy and lactation. This study provides information on the dam’s and the puppies’ successful treatment through breast milk during nursing.

## 2. Materials and Methods

A clinical case of *B. gibsoni* infection was analyzed in a 5-year-old pregnant American Pit Bull Terrier and her eight puppies (4 males and 4 females) during pregnancy and lactation.

From the dam and the viable puppies, non-clotted blood was collected from the jugular vein into an EDTA tube (1 mL of blood) and a tube without anticoagulants (2–3 mL of blood) for biochemical, hematological, and molecular analyses.

One male puppy was euthanized 10 days after birth due to poor health conditions. Several internal organs were dissected and preserved in tubes with 70% ethanol for further analysis. The liver, kidney, and spleen tissue were used to extract genomic DNA and for molecular analyses.

### 2.1. Hematological and Biochemical Analyses

A complete hematological analysis was evaluated with a ProCyte Dx analyzer (IDEXX Laboratories, Westbrook, ME, USA). Serum biochemical samples were processed with a Cobas c111 analyzer (Roche, Basel, Switzerland).

The biuret method measured total protein concentrations using commercially available diagnostic kits (Randox, Crumlin, UK) and an Alizé automated chemistry analyzer (Lisabio, Poully en Auxois, France). Separation and distribution of serum protein fractions were performed by zone electrophoresis on agarose gel using a Hydrasys automated electrophoresis system with commercial Hydragel 7 Proteine diagnostic kits (Sebia Corporate, Lisses, France). Protein fractions were quantified (g/L) from total protein concentrations according to optical density and percentage distribution of individual fractions on the electrophoretogram. Albumin/globulin (A/G) ratios were also calculated.

### 2.2. Detection of Antibodies against Pathogens, Molecular Detection of Pathogens, and Genetic Analyses

Genomic DNA was extracted from up to 10 mg of tissue samples (euthanized puppy) and 200 μL of EDTA-treated blood samples (dam and viable puppies) using a commercial DNA extraction kit (GeneJET Genomic DNA Purification Kit, Thermo Scientific, Waltham, MA, USA). For molecular detection of *Babesia* spp. and *B. gibsoni*, PCR amplifications of an approximately 450 bp fragment of the 18S rRNA gene, flanked by the reverse BJ1 (5′GTCTTGTAATTGGAATGATGG3′) and forward BN2 (5′TAGTTTATGGTTAGGACTACG3′) primers, and a 671 bp long fragment of species-specific 18S rRNA gene using primers Gib599F (5′CTCGGCTACTTGCCTTGTC3′) and Gib1270R (5′GCCGAAACTGAAATAACGGC3′), were performed, respectively [17,18].

Additionally, the dam was tested for concurrent infections such as *Mycoplasma* spp., *Hepatozoon canis*, *Anaplasma* spp., and *Dirofilaria* spp. by genus-specific or species-specific PCRs.

Sequenced DNA from *Babesia* spp. and *B. gibsoni*-positive dogs was used as a positive control in each PCR reaction, and nuclease-free water was added to the template as a negative control.

All *B. gibsoni*-positive PCR products were purified using a purification kit (Qiagen, Hilden, Germany) and sequenced in both directions. Nucleotide sequences were manually edited in MEGA X [19] and further compared to GenBank records using BLAST [20].

The presence of antibodies against *Borrelia burgdorferi* and *Ehrlichia canis* was examined by SNAP 4Dx^®^ Plus, IDEXX (Westbrook, ME, USA).

### 2.3. Statistical Analysis

The software package GraphPad Prism 8.0 (GraphPad, San Diego, CA, USA) was used for statistical analyses.

### 2.4. Administered Therapy

In the bitch diagnosed with *B. gibsoni* infection, therapy for 10 days with a combination of Malarone^®^ (13.5 mg/kg of atovaquone PO q24 h, with fatty meal), azithromycin (10 mg/kg PO q24 h), and artesunate (12.5 mg/kg PO q24 h) was initiated according to the protocol in Karasová et al. [21].

## 3. Results

### 3.1. Babesia gibsoni Infection in Dam

In a 19.3 kg pregnant bitch, markedly pale pinkish mucous membranes were observed 14 days before giving birth. The physical and clinical conditions of the female were appropriate for the degree of pregnancy; there was no loss of muscle mass, and no other clinical signs were manifested. Clinical examination revealed no other changes during pregnancy and at the delivery time, apart from pale mucous membranes.

The PCR tests for *Mycoplasma* spp., *Anaplasma* spp., *H. canis*, and *Dirofilaria* spp., as well as the tests for the presence of antibodies against *B. burgdorferi* and *E. canis*, were negative. However, on day 46 of pregnancy, the female was diagnosed with *B. gibsoni* infection via species-specific PCR.

#### 3.1.1. Clinical Manifestation of Infection before Initiation of Therapy

Hematological and biochemical changes on day 46 of pregnancy (14 days before delivery) and 3 days after delivery showed the occurrence of non-regenerative anemia, hyperproteinemia, significant hypoalbuminemia, and a reduced value of the A/G ratio (Table 1). The values of the white blood count and other biochemical indicators (ALT, ALP, Crea, Urea, AMS, and bilirubin) were within the reference range during the entire follow-up; therefore, they are not included in the table.

The condition of the dam began to deteriorate 28 days after delivery. She had lost 0.7 kg in the last seven days and had noticeably pale pinkish mucous membranes. Laboratory blood tests revealed that the anemia had further worsened. Consequently, therapy was initiated according to the therapeutic protocol by Karasová et al. [21].

#### 3.1.2. Clinical Manifestation of Infection after Initiation of Therapy

**Scheme 1:**
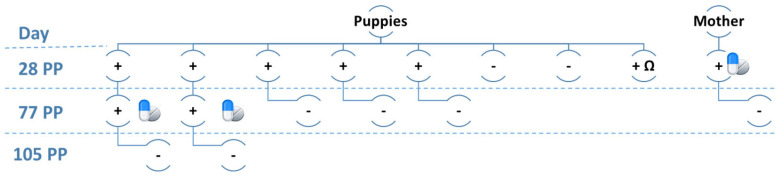
Graphic presentation of the PCR results in dam infected by *B. gibsoni* and her puppies during monitoring. PP, postpartum; +, *B. gibsoni* PCR-positive result; −, PCR-negative result; Ω, euthanasia. The beginning of the therapy is represented by a picture of the medication.

A control PCR examination on day 42 after delivery (14 days from the start of therapy), as well as on day 77 after delivery (49 days from the start of therapy), revealed negative results for the presence of *B. gibsoni* (Scheme 1).

Hematological and biochemical parameters from the days with a positive PCR result were statistically averaged (mean ± SD) and compared with those measured on days 42 and 77 after delivery (PCR-negative results). The values of red blood cells, hemoglobin concentration, and hematocrit increased on day 42 after delivery, and on day 77 they were already within the reference range. A significant increase in reticulocytes compared to the mean value at the time of PCR positivity occurred on day 77 after delivery (*p* < 0.001) simultaneously with an increased level of platelets (*p* < 0.01). Even though the A/G ratio was still low on day 77, its value increased compared to the average value at the time of PCR positivity, and on day 77 after birth, it was already just below the lower limit of the norm. Total protein reached the reference range on day 42 postpartum, and albumin values, despite a significant increase, were just below the lower limit of normal on day 77 postpartum.

#### 3.1.3. Protein Fractions Alterations

**Figure 1:**
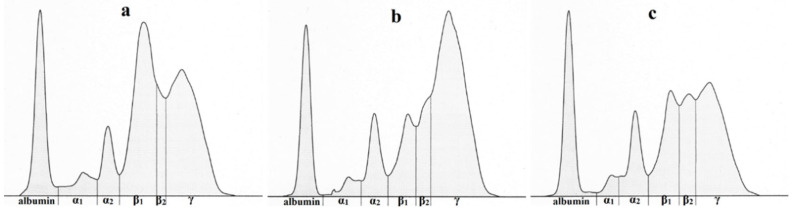
Electrophoretograms of the *B. gibsoni* infected dam 14 days before (**a**), 3 days after (**b**), and 28 days after parturition (**c**).

Six protein fractions were identified on the serum protein electrophoretogram of the bitch, including albumin, α1-, α2-, β1-, β2-, and γ-globulins, but the size and shape of separated serum protein fractions differed according to the stage of the peripartal period (Figure 1a–c). On day 14 before delivery, β- and γ-globulins were the most prominent fractions and were characterized by high and wide peaks on the electrophoretogram, but the β2-globulin zone was not clearly demarcated from the β1-globulin fraction. The albumin fraction showed a sharper electrophoretic amplitude, while the α-globulins were comparable to those observable in healthy dogs. Electrophoresis on day 3 postpartum showed marked changes, especially in the β and γ zones. The γ-globulin fraction increased markedly, showing a very high (higher than albumin) and wide peak, and constituted approximately 50% of total serum proteins. Furthermore, β-γ bridging with no clear valley between these zones was observable. On day 28 after parturition, the β- and γ-globulins started to decrease, while the albumin fraction slightly increased. The β-globulins were separated into two distinct bands, and the γ-globulin zone was also clearly demarcated from the β2-globulin fraction.

### 3.2. Clinical and Pathological Manifestation of B. gibsoni Infection in Puppies

Even though all puppies were born viable with a good sucking reflex, 12 h after birth one of them (a male) began to lose interest in sucking milk, had difficult defecation and urination, showed a distended and painful abdomen, and was not developing at the same rate as its siblings. A slight icteric discoloration of the sclera appeared on day 9 after birth. Despite the owner’s efforts to save the puppy, it had to be euthanized 10 days after birth, and was subjected to a pathological examination.

**Figure 2:**
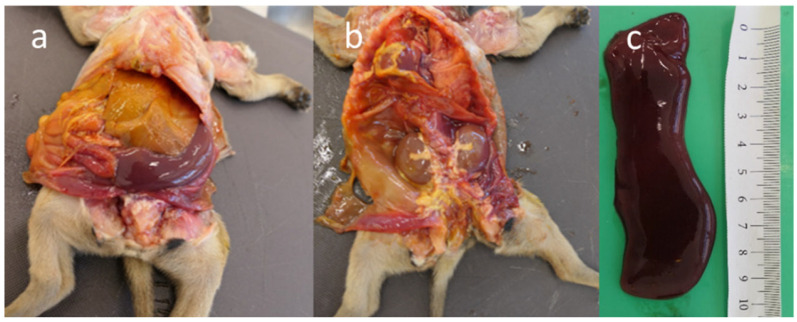
Pathological examination of euthanized puppy transplacentally infected with *B. gibsoni* showing splenomegaly and icterus of subcutaneous fat, liver, intestines, mesenteric fat (**a**), pericardial fat, liver, kidneys, and subcutaneous fat in the abdominal and pelvic regions (**b**). Splenic enlargement was the most significant pathological change observed (**c**).

During the necropsy, mild icteric staining of the kidneys, lungs, intestines, mesenteric fat, and subcutaneous fat in the abdominal and pelvic regions, as well as significant icteric staining of the liver and pericardial fat, was found in the euthanized puppy. Significant splenomegaly dominated the pathological changes (Figure 2).

All DNA samples extracted from the tissue samples taken from the spleen, liver, kidneys, heart, brain, and lungs tested PCR-positive for the presence of *B. gibsoni*, suggesting the pup’s pathological condition was caused by the manifestation of *B. gibsoni* infection acquired by transplacental transmission from a positive dam.

The other seven puppies underwent hematological and biochemical blood analysis at the ages of 28 and 77 days, simultaneously with PCR examinations for the presence of *B. gibsoni*. The presence of *B. gibsoni* was confirmed in five viable puppies on day 28 after birth (Scheme 1). Hematological and biochemical changes in the blood parameters of the puppies are shown in Table 2.

A control blood sample was taken from all puppies on day 77 after birth (49 days post initiation of dam’s treatment) and it was found that three out of five previously positive individuals already showed a negative PCR test for *B. gibsoni*. This suggests that three puppies were successfully treated via the dam’s milk during lactation (Scheme 1).

Anemia was not confirmed in any puppies of the investigated groups, although on day 77 the erythrocyte level was lower in the group of positive dogs than in the other two groups. Higher reticulocyte values were noted in positive dogs on day 77 compared to other groups. A statistically significant increase in the mean value of bilirubin (*p* ˂ 0.01) together with a decreased creatinine level (*p* ˂ 0.05) occurred only in the group of positive dogs on day 77 after birth. However, creatinine levels were below the norm in all groups on days 28 and 77, respectively. Positive dogs on day 77 also showed a lower value of creatine kinase compared to the other groups. Albumin concentrations were below the limit of the reference norm in both groups on day 28, but in the group of PCR-positive dogs, they were lower than in the group of PCR-negative dogs.

#### 3.2.1. Clinical Manifestation of Infection after Initiation of Therapy

Based on the positive results of the PCR examination, at the age of 77 days, two PCR-positive puppies (8.1 kg and 8.2 kg) were treated with a combination of Malarone^®^, azithromycin, and artesunate [21].

A follow-up PCR test on day 91 (14 days from the start of therapy) showed PCR-positive results in both puppies. Additionally, they underwent hematological and biochemical examination, and bilirubin values were 0.00 µmol/L in both dogs.

At the age of 105 days (28 days from the start of therapy), these two puppies tested PCR-negative for *B. gibsoni* (Scheme 1). All biochemical parameters, except creatinine (28.8 ± 1.22 μmol/L) and urea (2.22 ± 0.3 mmol/L), which remained below the reference level, were within the normal reference range 105 days after birth.

All seven puppies were repeatedly tested via PCR at six months of age and all tested negative for *B. gibsoni*.

#### 3.2.2. Protein Fractions Alterations

**Figure 3:**
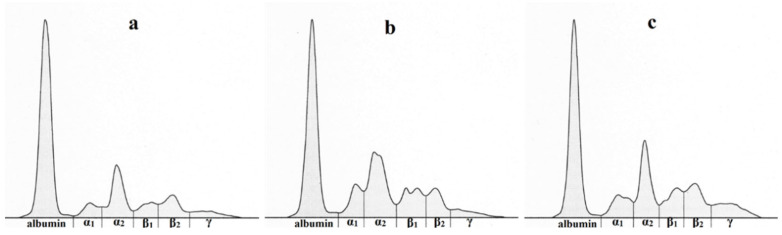
Representative electrophoretograms of a PCR-negative puppy (**a**) and a PCR-positive puppy on days 28 (**b**) and 77 of age (**c**).

Similarly, to the dam, serum proteins in puppies were separated into six fractions: albumin and α1-, α2-, β1-, β2-, and γ-globulins. Albumin was the most prominent fraction on the electrophoretogram in *B. gibsoni*-positive puppies, presenting a high and narrow peak. The α-globulins migrating into α1- and α2-subfractions were higher than those observable in PCR-negative puppies. The α2-globulin fraction tended to show a twin peak on day 28 of age. The β-globulin zone was separated into β1- and β2-subfractions, and on day 28 of age, the β1-globulin fraction showed a double peak with two approximately similarly sized moderate peaks. The γ-globulin fraction was presented as a low and flat zone, which increased on day 77 of age (Figure 3a–c).

### 3.3. Nucleotide Sequences Obtained in This Study

*Babesia gibsoni*-positive PCR amplicons obtained at the beginning of the screening were further sequenced. Seven nucleotide sequences were manually edited and compared to GenBank records using BLAST. The sequences of 18S rRNA from the female and puppies were identical in overlapping regions and have shown 99.68–100% similarity with isolates from Asia, such as, e.g., the Nanjing0009 strain (HG328235) from China, Chacha (OQ716579), Wayanad isolate 1 (MN134511), Trivandrum isolate 1 (MN134509), and Trissuris 2 (MN134508) from India. All nucleotide sequences of the 18S rRNA gene fragment of *B. gibsoni* have been submitted to the GenBank database and are available under the following accession numbers: dam OQ857321, positive puppies OQ857322-26, and euthanized puppy OQ857327.

## 4. Discussion

The transplacental transmission of *B. gibsoni* from an infected dam to unborn puppies contributes to the spread of the parasite within canine populations. In 2005, the transplacental transmission of *B. gibsoni* was confirmed during an experimental infection of a bitch [13]. All newborn puppies were infected and died a few days after birth. In another clinical study, a case of pregnancy of a *B. gibsoni*-positive female, who was asymptomatic during the entire follow-up, was described. She gave birth to six puppies and two of them showed severe anemia (on days 8 and 29). Subsequently, both (2/6; 33%) were diagnosed with *B. gibsoni* by PCR. These puppies received blood transfusions and were treated with high doses of clindamycin. The other four puppies tested negative (4/6; 67%) [25]. According to Kirk [15], in bitches who tested positive for *B. gibsoni* at the time of pregnancy and delivery, the probability of positive newborn puppies is 25–80%, and they reach high levels of pup mortality (25–100%), stillbirth, or neonatal death. Similarly, in our clinical case study, one out of eight puppies had to be euthanized due to their poor clinical condition at the age of 10 days. The puppy’s vitality gradually deteriorated, probably related to severe hemolysis caused by high parasitemia. Through the pathological examination of the euthanized puppy, severe icterus of the internal organs, like in the study by Fukumoto et al. [13], was observed. Moreover, significant hepatomegaly was present. *Babesia gibsoni* was confirmed by PCR examination of the tissues taken at autopsy, suggesting that the severe clinical changes related to infection caused the death. Another five puppies were identified as positive at the first examination (28 days after birth) and thus the total incidence of *B. gibsoni* in this progeny reached 75% (6/8).

Kirk et al. [15] used the combined treatment of Malarone^®^ with azithromycin in the therapy of *B. gibsoni*, and claimed that if females are treated during pregnancy the incidence of the birth of infected puppies is significantly reduced, and the risk of transplacental transmission decreases to zero. In our case, the dam was treated on day 28 after parturition; as her anemia worsened, her appetite deteriorated and she became lethargic. The clinical condition of the dam, as well as the laboratory parameters, improved on day 77 after treatment with the therapeutic protocol by Karasová et al. [21] using administration of the combination of Malarone^@^, azithromycin, and artesunate. The PCR test for the presence of *B. gibsoni* brought negative results on day 42 after delivery (14 days from the start of the therapy). The clinical and laboratory findings also correlated with the changes observable in serum protein electrophoretic pattern when the extremely high peaks in the β- and γ-globulin zones started to decrease, reflecting a positive response to treatment.

Kirk et al. [15] tested puppies from a *B. gibsoni*-infected dam for the first time at the age of 4 weeks by PCR and started with treatment in positive puppies at 10 weeks of age because they did not respond sufficiently to therapy earlier. The dam of the pups in our study underwent therapy on day 28 postpartum and nursed all pups during therapy. At the first examination at the age of 28 days, we identified five positive puppies, but the repeated examination on day 77 showed a positive PCR result only in two puppies. This means that parasitemia was eliminated in three puppies that were not treated individually by any medication. This fact points to the presence of not only the effective drug concentrations in breast milk but also the possible immunostimulant effect of breast milk.

According to the published information, it was found that low concentrations of artesunate and Malarone^®^ pass into breast milk, but they are not sufficient to protect newborns against malaria [26]. Similarly, azithromycin is excreted in human milk, but due to the slow clearance and accumulation of azithromycin, these levels are difficult to interpret. The dose of antibiotics that the infant would receive in milk gradually increases over several days as the levels of the drug also increase in the mother’s blood until a steady state is reached, which takes about three days [27,28]. After oral administration, artesunate is relatively rapidly metabolized to DHA, which is much more effective than the original substance and contributes to a greater extent to the overall antimalarial activity [29].

The mean absolute oral bioavailability of artesunate in acute malaria patients was 61% [30]. After a single oral dose of 200 mg of artesunate to lactating mothers, the substance was not detectable in breast milk (<5 µg/L), but its active metabolite DHA reached a maximum concentration of approximately 35 mg/L at 90 min post dose in breast milk and at 6 h post dose it was no longer detectable (<2.5 mg/L) [31]. Based on these studies, we assume that in the three cured puppies, the administered drugs and DHA were present in the dam’s milk in a sufficient concentration to eliminate the infection in the puppies, considering their low weight.

Our hypothesis is based on the results of clinical studies in lactating women, as information on the pharmacokinetics of these drugs in lactating pet animals is currently unavailable. Many experimental studies describe the immunomodulatory effects of artesunate on the innate and adaptive components of immunity in autoimmune, infectious, and oncological diseases [32].

Similar to malaria in humans, babesiosis in animals is considered not only a parasitic disease but also an immune-related disease with an excessive inflammatory response, especially the overproduction of pro-inflammatory cytokines and chemokines and the involvement of the parasite in the activation of macrophages [33]. Pro-inflammatory cytokines and chemokines, especially tumor necrosis factor alpha (TNF-α), interferon-gamma (IFN-γ), and interleukins IL-6 and IL-8 (also known as CXCL8) contribute to the pathogenesis of the disease and an intense pro-inflammatory immune response, in addition to IL-12, IL-18, monocyte chemoattractant protein 1 (MCP-1, also known as CCL2), and others. In addition, another manifestation of the disease is the insufficient and/or delayed production of anti-inflammatory cytokines, such as IL-4 and IL-10 [34,35,36,37,38]. Several studies have confirmed that artesunate inhibits the signaling pathway of the transcription factor NF-κB, leading to reduced synthesis and secretion of pro-inflammatory cytokines in monocytes and macrophages. In a mouse model of sepsis, this substance inhibited the production of TNF-α and IL-6 in peritoneal macrophages in a dose-dependent manner by inhibiting the expression of Toll-like receptor 2 (TLR2) and suppressing the activation of NF-κB [39]. In the case of experimental cerebral malaria, the authors found a higher mRNA expression of the pro-inflammatory cytokines IL-2, IL-6, IL-10, IL-17, IFN-γ, and TNF in the serum, hippocampus, and frontal cortex in mice infected with *Plasmodium berghei*. A single dose of artesunate therapy reduced the expression of these pro-inflammatory cytokines, while a significant improvement in neurological signs and survival was observed in treated mice [40].

Based on studies on the suppression of pro-inflammatory cytokine production, knowledge of adverse effects, and other markers when using artesunate in patients with malaria, a similar immunomodulatory anti-inflammatory effect can be expected in puppies with *B. gibsoni* infection. However, to confirm our hypothesis, it would be necessary to expand the study by determining immune markers in the serum of the dam and puppies.

The monitored hematological and biochemical parameters of the puppies did not show remarkable differences between the groups. More significant changes were seen in the group of PCR-positive dogs on day 77 and consisted of a lower count of erythrocytes, a significantly higher level of bilirubin, and a significantly lower level of creatinine compared to the other two groups (negative and negative without therapy). A lower concentration of erythrocytes, hemoglobin, and hematocrit could indicate incipient anemia caused by hemolysis, resulting in a higher bilirubin concentration. Low creatinine values may be due to loss of muscle mass, although puppies generally have low creatinine concentrations [23,24]. In all groups of dogs, creatinine levels were decreased compared to the reference norm. Still, there are only a few studies dealing with establishing norms for hematological and biochemical parameters in puppies at the age of several weeks, and the values in these studies differ somewhat, mainly depending on the breed. In a study by Fukumoto et al. [13], positive pups suffered from severe clinical signs including anemia. However, in this study, the infection of the dam was experimental, and the clinical condition of the puppies probably also depended on the dose to which the dam was exposed. Our positive puppies did not show any clinical signs of the disease, nor did the laboratory parameters show significant differences between the groups of positive and negative puppies. In PCR-positive puppies, alterations were observed on the serum protein electrophoretogram with higher peaks in the α1- and α2-globulin zones compared to PCR-negative puppies, which could probably be attributed to the activation of inflammatory responses in these animals. Similarly, in the study conducted by Brown et al. [41], experimental *B. gibsoni* infection of beagle puppies induced a marked but delayed acute-phase response, characterized by a marked increase in the concentrations of C-reactive protein.

The therapeutic protocol by Karasová et al. [21] was used in two puppies that showed positivity on day 77, and negativity during the next control PCR examination on day 105. At the same time, hematological and biochemical parameters improved, except for persistently lower creatinine and urea concentrations. Since we did not carry out laboratory blood tests for the other puppies, it is not possible to compare the values of the kidney parameters between the groups. However, we assume that these values would also be similar for the other puppies if we proceed from the previous samplings, where the creatinine and urea concentrations were equally low in all groups.

All seven puppies were retested by PCR at six months of age for the presence of *B. gibsoni*, and in all cases the result was negative, including puppies that were cured through the dam’s milk.

## 5. Conclusions

Transmission of drugs through breast milk is a complex topic that depends on various factors such as the properties of the drug, the timing of drug administration, the dosage, the dam’s metabolism, the offspring’s age and health, and more.

This study confirmed that three of five puppies transplacentally infected with *B. gibsoni* were successfully treated through therapy administered to the nursing dam and the other two puppies were treated by same protocol as the dam. Treatment was safe for both dam and puppies in terms of adverse effects.

## Figures and Tables

**Table 1:** Hematological and biochemical values in bitch infected with *B. gibsoni* in the period before and after parturition.

		PCR-Positive				PCR-Negative	
Parameter	Reference Range	14 AP	3 PP	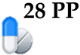	PCR-Positive Mean ± SD	42 PP	77 PP
RBC, 10^12^/L	5.65–8.87	4.02 •	3.6 •	3.5 •	3.71 ± 0.28 •	3.9 •	5.8
HCT, %	37.3–61.7	25.2 •	23.3 •	22.2 •	23.57 ± 1.51 •	24.1 •	37.4
HGB, g/dL	13.1–20.5	8.3 •	7.7 •	7.5 •	7.83 ± 0.42 •	7.9 •	13.2
RET, K/µL	10.0–110.0	30.6	76.2	30.5	45.77 ± 26.36	61.8	160.9 *** •
PLT, K/µL	148–484	188	165	180	177.67 ± 11.68	149	223.00 **
TP, g/L	47–74	97.7 •	84.7 •	76.9 •	86.43 ± 10.51 •	71.8	69.7
ALB, g/L	26–41	15.1 •	11.9 •	14.2 •	13.73 ± 1.65 •	16.8 •	24.2 •
A/G	0.6–1.1	0.18 •	0.16 •	0.23 •	0.19 ± 0.04 •	0.3 •	0.53 •

The data from days 14 AP, 3 PP, and 28 PP are processed as mean ± SD. Significance of 42 PP and 77 PP versus PCR-positive mean ± SD: ** *p* < 0.01; *** *p* < 0.001. Values outside the reference range are marked as •; AP, antepartum; PP, postpartum; the beginning of the therapy is represented by a symbol of the medication. RBC, red blood cells; HCT, hematocrit; HGB, hemoglobin; RET, reticulocyte count; PLT, platelet count; TP, total protein; ALB, albumin; A/G, albumin/globulin ratio.

**Table 2:** Comparison of hematological and biochemical parameters in seven puppies of *B. gibsoni*-infected dam on days 28 and 77 after birth.

	Day 28 PP			Day 77 PP			
	Reference Range	Mean ± SD		Reference Range	Mean ± SD		
Parameter		PCR + (*n* = 5)	PCR − (*n* = 2)		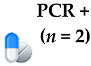	PCR- No Therapy (*n* = 3)	PCR − (*n* = 2)
RBC, 10^12^/L	3.2–4.6	3.92 ± 0.43	3.91 ± 0.01	3.9–5.7	4.62 ± 1.60	5.38 ± 0.11	5.01 ± 0.18
HCT, %	23–33	26.60 ± 1.36	26.60 ± 0.42	26–38	31.40 ± 7.21	33.70 ± 1.64	32.20 ± 0.84
HGB, g/dL	7.0–10.8	8.26 ± 0.75	8.25 ± 0.21	8.2–11.8	10.00 ± 3.11	11.33 ± 0.40	10.75 ± 0.21
RET, K/µL	29–678	292.80 ± 194.30	213.50 ± 38.33	91–384	218.55 ± 231.86	75.60 ± 19.05 •	44.00 ± 1.13 •
PLT, K/µL	199–736	319.40 ± 128.30	368.00 ± 39.60	173–714	224.50 ± 235.47	360.00 ± 21.17	308.50 ± 13.44
WBC, 10^9^/L	8.3–20.0	16.26 ± 2.89	19.99 ± 1.56	8.4–25.2	16.79 ± 4.61	17.87 ± 5.57	13.97 ± 13.95
NEU, 10^9^/L	4.0–12.8	8.31 ± 2.55	9.43 ± 0.63	3.6–14.2	10.30 ± 3.49	11.17 ± 4.85	8.03 ± 0.16
LYM, 10^9^/L	2.5–8.9	5.38 ± 0.89	7.65 ± 0.83	2.4–12.4	4.87 ± 1.20	5.03 ± 0.71	4.73 ± 0.02
MONO, 10^9^/L	0.5–1.5	1.87 ± 0.23 •	2.16 ± 0.04 •	0.5–2.1	1.13 ± 0.35	1.09 ± 0.20	0.96 ± 0.01
EOS, 10^9^/L	0.1–1.1	0.69 ± 0.14	0.63 ± 0.21	0.0–2.2	0.49 ± 0.28	0.57 ± 0.19	0.25 ± 0.21
BASO, 10^9^/L	ND	0.01 ± 0.01	0.13 ± 0.16	ND	0.02 ± 0.01	0.01 ± 0.01	0.01 ± 0.00
ALT, µkat/L	0.2–0.72	0.30 ± 0.03	0.32 ± 0.03	0,3–0,56	0.22 ± 0.01 •	0.28 ± 0.80 •	0.21 ± 0.04 •
T BIL, µmol/L	0.2–2.3	0.16 ± 0.22	0.00 ± 0.00	0,2–2,3	1.00 ± 0.85 **	0.00 ± 0.00	0.20 ± 0.28
AMS, µkat/L	4.6–19	4.87 ± 0.35	4.66 ± 0.16	4,85–16,73	3.35 ± 1.08 •	4.56 ± 1.80 •	3.01 ± 0.14 •
CREA, µmol/L	32–65	17.22 ± 9.39 •	23.10 ± 1.98 •	34–63	3.35 ± 1.06 * •	9.97 ± 6.96 •	8.35 ± 0.92 •
UREA, mmol/L	2.2–6.4	3.14 ± 0.34	3.13 ± 0.33	2,0–5,6	1.23 ± 0.30 •	1.35 ± 0.43 •	1.09 ± 0.09 •
CK, μkat/L	3.65–14.05	7.54 ± 1.91	7.30 ± 0.14	2,75–11,7	2.60 ± 0.42 •	4.20 ± 0.98	3.20 ± 0.28
TP, g/L	34–47	34.48 ± 1.78	35.95 ± 0.21	36–48	50.45 ± 3.89	50.00 ± 2.19	47.45 ± 0.07
ALB, g/L	18–30	15.86 ± 1.32 •	17.70 ± 0.85 •	19–32	25.25 ± 0.64	23.33 ± 1.79	23.50 ± 0.99
Alpha 1, g/L	ND	3.16 ± 0.38	2.65 ± 0.21	ND	4.30 ± 0.71	4.23 ± 0.15	4.00 ± 0.71
Alpha 2, g/L	ND	7.26 ± 0.62	7.65 ± 0.64	ND	7.25 ± 0.07	7.78 ± 0.55	6.95 ± 0.78
Beta 1, g/L	ND	4.06 ± 0.32	3.80 ± 0.00	ND	5.05 ± 0.07	4.47 ± 0.59	4.20 ± 0.14
Beta 2, g/L	ND	2.88 ± 0.22	2.95 ± 0.21	ND	4.60 ± 0.42	5.83 ± 0.32	5.00 ± 1.13
Gamma, g/L	ND	1.26 ± 0.32	1.20 ± 0.00	ND	4.05 ± 2.19	4.30 ± 0.46	3.85 ± 0.07
A/G	ND	0.85 ± 0.06	0.98 ± 0.11	ND	1.01 ± 0.11	0.88 ± 0.06	0.98 ± 0.08

Data are expressed as mean ± SD. Significance of PCR-positive versus PCR-negative in the same day: * *p* < 0.05; ** *p* < 0.01. Values outside the reference range are marked as •; ND, values not determined; PP, postpartum; PCR +, positive PCR finding of *B. gibsoni*; PCR −, negative PCR finding of *B. gibsoni*; RBC, red blood cells; HCT, hematocrit; HGB, hemoglobin; RET, reticulocyte count; PLT, platelet count; WBC, white blood cells; NEU, neutrophils; LYM, lymphocytes; MONO, monocytes; EOS, eosinophils; BASO, basophils; ALT, alanine aminotransferase; T BIL, total bilirubin; AMS, amylase; CREA, creatinine; CK, creatine kinase; TP, total protein; ALB, albumin; Alpha 1, Alpha 2, Beta 1, Beta 2, Gamma globulins; A/G, albumin/globulin ratio. The beginning of the therapy is represented by a symbol of the medication. Reference ranges adapted according to the studies in [22,23,24].

## Data Availability

All data are provided in the article.
